# Prevalence of IncFIB Plasmids Found among *Salmonella enterica* Serovar Schwarzengrund Isolates from Animal Sources in Taiwan Using Whole-Genome Sequencing

**DOI:** 10.3390/pathogens10081024

**Published:** 2021-08-13

**Authors:** I-Chen Li, Hsiu-Hui Wu, Zeng-Weng Chen, Chung-Hsi Chou

**Affiliations:** 1 Zoonoses Research Center, School of Veterinary Medicine, National Taiwan University, No. 1, Sec. 4, Roosevelt Road, Taipei City 106, Taiwan; d06629004@ntu.edu.tw; 2 Animal Technology Research Center, Agricultural Technology Research Institute, No. 52, Kedong 2nd Road, Zhunan Township, Miaoli County 350, Taiwan; hhwu@mail.atri.org.tw

**Keywords:** *Salmonella*, whole-genome sequencing, baseline data, IncFIB

## Abstract

*Salmonella enterica* serovar Schwarzengrund is one of the most frequently isolated *Salmonella* serotypes responsible for human and poultry infections in Taiwan, and it has raised public health concerns. To better facilitate the understanding of transmission patterns and the dynamics of epidemics, sharing molecular data on pathogen profiles is urgently needed. The objectives of the current study were to determine and establish baseline data of *S. enterica* serovar Schwarzengrund isolates from 23 epidemiologically unrelated sources from year 2000 to 2018 and examine their phenotypic and genotypic characteristics. Genomic DNA of the *Salmonella* isolates was extracted and subjected to whole-genome sequencing using an Illumina platform. Results showed that all selected isolates exhibited multidrug resistance, and six of those were resistant to ciprofloxacin phenotypically. Genotypically, these isolates carried genes resistant to aminoglycoside (100%), phenicol (91.3%), β-lactams (69.5%), folate pathway antagonist (100%), tetracycline (82.6%), and fluoroquinolone (4.3%). Moreover, these isolates harbor integrons with five different gene cassettes identified for the first time, which are associated with resistance to trimethoprim, streptomycin, tetracycline, sulfonamide, chloramphenicol, and gentamicin. Furthermore, prevalence of IncFIB plasmid was found among studied isolates, which may increase its ability to colonize the chicken cecum and cause extra-intestinal disease. *Salmonella* pathogenicity islands SPI-1 to SPI-5, SPI-13, and SPI-14, as well as C63PI locus, were also detected in all isolates. This study demonstrated that a considerable high antimicrobial resistance with high virulence levels of *Salmonella* were found from animal sources. Sharing data on these pathogen profiles can not only help increase the reproducibility and accessibility of genomic analysis but can also support surveillance and epidemiological investigations for salmonellosis in the region.

## 1. Introduction

Throughout history, the emergence and reemergence of infectious diseases remain major causes of morbidity and mortality worldwide [1]. In 2016, infectious diseases killed approximately 10 million people, accounting for one-fifth of all deaths worldwide [2]. Even today, the world continues to confront old diseases such as salmonellosis and tuberculosis, as well as new diseases such as Ebola and COVID-19. Since infectious diseases are unavoidable in life, preventive strategies should be developed to control and mitigate the transmission of infections.

Insights into the genomes of infective organisms are paramount in disease prevention, management, and treatment. From the 1990s, public health authorities and food regulators started applying pulsed-field gel electrophoresis (PFGE) molecular subtyping for surveillance and outbreak investigations [3]. Before the nationwide routine use of PFGE, only five outbreaks (a mean of 54 cases per outbreak) of listeriosis with an identified source were solved over 14 years [4]. However, after five years of routine PFGE usage, eleven outbreaks with a median of five cases per outbreak were identified [4]. Although PFGE has proven remarkably useful in detecting listeriosis clusters and other pathogens such as *Salmonella* [5] and *E. coli* [6], it has some limitations. Genomic insertions, deletions, rearrangement, and point mutation at the restriction enzyme sites can cause misinterpretation of the PFGE results, which may hamper or delay the discovery of an outbreak. Moreover, the PFGE database is a closed system, as only network participating laboratories can have access to it.

On the other hand, when compared to PFGE, the emergence of affordable whole-genome sequencing (WGS) technologies, along with the development of sophisticated bioinformatics analytical tools, offers a much finer resolution, as it captures DNA sequence changes across the entire genome of single microbial isolates [7]. WGS data are inherently digital, standardized, and can be accessed at any time by the general public, while PFGE data require standardized protocols to make inter-laboratory comparisons of DNA patterns [8]. Statistics also showed that, after 2 years of transition from PFGE to WGS for outbreak investigation, improvements in the number of clusters detected and outbreaks solved, with a marked reduction in median cluster size, were observed [9]. Due to these advantages, a 100 K Pathogen Genome Project launched in 2012 to sequence 100,000 pathogen genomes for use in host–microbe interactions, public health, and genome ecology [10]. To date, public health agencies have used pathogen genomics in almost every infectious disease program for surveillance and epidemiological investigations [11].

Raw sequence data can be stored in the sequence read archive (SRA) at the National Center for Biotechnology Information (NCBI) of the US National Institutes of Health (NIH) [12]. From this aspect, this approach laid a foundation for the globalization of pathogen surveillance. Hence, to better facilitate the understanding of transmission patterns and the dynamics of epidemics, sharing molecular data on pathogen profiles is urgently needed. Among many *Salmonella enterica* serovars, serovar Schwarzengrund is one of the most frequently isolated *Salmonella* serotypes responsible for human and poultry infections [13]. In Taiwan, *S. enterica* serovar Schwarzengrund with high resistance to ampicillin, gentamicin, kanamycin, streptomycin, tetracycline, nalidixic acid, trimethoprim-sulfamethoxazole, and chloramphenicol was found to be the most prevalent serotype (30.5%) in raw chicken meat [14]. In Japan, the percentage of *S. enterica* serovar Schwarzengrund highly resistant to streptomycin, sulfamethoxazole, and oxytetracycline was found to steadily increase from 2.1 in 2009–2012 to 21.3 in 2013–2016 in broiler chickens [15]. This increase in the incidence of *S. enterica* serovar Schwarzengrund in food is considered a threat, as previous studies have shown that multi-drug-resistant *S. enterica* serovar Schwarzengrund could spread internationally from imported contaminated food products to persons in Denmark and the United States [16]. In addition, resistance genes may also spread from animals to humans via mobile genetic elements such as plasmids and integrons [17]. However, there is scarce information on the role of resistance plasmids in the spread of multi-drug-resistant *Salmonella*, particularly *S. enterica* serovar Schwarzengrund. Hence, the objectives of the current study were to determine and establish baseline data of *S. enterica* serovar Schwarzengrund isolates from 23 epidemiologically unrelated sources from year 2000 to 2018 and examine their phenotypic and genotypic characteristics.

## 2. Results

### 2.1. Genome Size and Characteristics

The total length of the final assemblies of 23 *S. enterica* serovar Schwarzengrund strains ranged from 4.64 to 5.02 Mb (Table 1). These assemblies produced between 12 and 201 contigs, with a GC content found between 51.8 and 52.2%. Genome annotation using RAST identified 4668 to 5176 coding sequences and 84 to 104 RNAs genes, distributed in 363 to 371 subsystems (Table 1), indicating about 3% variation among the *S. enterica* serovar Schwarzengrund genomes.

Genes assigned to different subsystems are summarized in Table 2. Among 27 subsystems, 9 subsystems (“photosynthesis”, “miscellaneous”, “nucleosides and nucleotides”, “cell division and cell cycle”, “motility and chemotaxis”, “secondary metabolism”, “fatty acids, lipids, and isoprenoids”, “nitrogen metabolism”, “sulfur metabolism”) were identified with a similar number of genes within the 23 *S. enterica* serovar Schwarzengrund isolates (data not shown). The most abundant subsystems were amino acids and derivatives metabolism (ranging from 339 to 343 genes), followed by carbohydrate metabolism (ranging from 334 to 339 genes), and protein metabolism (ranging from 216 to 231 genes). Regarding virulence factors, 9 to 26 genes and 51 to 64 genes were annotated as belonging to the “phages, prophages, transposable elements, plasmids” and “virulence, disease and defense” categories, respectively. 

### 2.2. Phenotypic and Genotypic Antimicrobial Resistance

Phenotypically, all isolates examined in this study exhibited multidrug resistance (resistant to three or more classes of antimicrobial), and six of those were resistant to ciprofloxacin, the first-line drug for treating *Salmonella* infections (Table 3). Resistance genes were observed for aminoglycoside (*aac*(6’)-Iaa, *aac*(3)-IV; 100%), phenicol (*cml*A1, *cat*A2, *floR*; 91.3%), β-lactams (*bla*_TEM-1B_; 69.5%), folate pathway antagonist (*sul*1, *sul*2, *sul*3, *dfr*A12; 100%), tetracycline (*tet*(A), *tet*(M); 82.6%), and fluoroquinolone (*aac*(6’)-Ib-cr; 4.3%). The most common resistance gene identified was an aminoglycoside acetyltransferase gene *aac*(6′)-I-aa, which was present in all isolates. The second most prevalent genes were *cml*A and *dfr*A, which confer resistance to chloramphenicol and trimethoprim, respectively. The phenotypic resistance correlated with the presence of antimicrobial resistance genes identified in 69.5% of the cases. It is worth noting that two isolates showed no resistance to chloramphenicol but carried *cml*A1 resistance genes. Moreover, 2 and 5 of the 23 *S. enterica* serovar Schwarzengrund isolates, predicted to be ampicillin- and ciprofloxacin-susceptible, respectively, were resistant.

### 2.3. Plasmids, Integrons, and Salmonella Pathogenicity Islands Analysis

A total of 7 plasmid types were observed in 21 of the 23 *S. enterica* serovar Schwarzengrund isolate strains, including IncF, IncH, IncX, IncL, IncI, IncQ, and Col (Table 4). According to Plasmid Finder, 43.4% (10/23) of isolates carried at least one plasmid, while 47.8% (11/23) of isolates carried two or more plasmid types. The dominant incompatibility group was IncFIB, followed by IncQ, Col440I, and Col440II. Additionally, IncHI2, IncHI2A, Col(pHAD28), Col156, Col(BS512), IncX1, IncL, IncI2, IncFII(pCTU2), and IncI1-I(Alpha) were detected at lower levels.

Using an Integron Finder followed by BLASTp analysis, a total of 22 complete integrons belonging to class I were found in 23 isolates (Table 4). At least two (*n* = 1), three (*n* = 5), four (*n* = 13), and up to five attC (*n* = 3) sites were found in a single isolate (data not shown). Among these integrons, six different gene cassette arrays, including *dfr*A12*-aad*A*-cml*A*-sul*3, *dfr*A12*-aad*A*-cml*A, *dfr*A12*-aad*A, *aad*A*-cml*A*-sul*3, *dfr*A12*-aad*A*-cml*A*-tet*R*-tet*(A), and *dfr*A12*-aad*A*-aac*(6’)-Ib-cr*-cml*A*-sul*3, were identified in the isolates (Table 4). 

Regarding the virulence capability of these isolates, eight SPIs (SPI-1, SPI-2, SPI-3, SPI-4, SPI-5, SPI-9, SPI-13, SPI-14), as well as centisome 63 pathogenicity island (C63PI), were detected in all genomes.

## 3. Discussion

As whole-genomic sequencing technologies have become affordable in recent years, these technologies are rapidly gaining acceptance as routine methods, and are transforming laboratory procedures [18]. As such, collection and sharing of genetic data is urgently needed, as it can provide more accurate bacterial identification, more robust phylogenetic relationships, and more definitive answer for epidemiological investigations. Hence, for the first time, 23 *S. enterica* serovar Schwarzengrund isolates were completely sequenced in this study to examine their phenotypic and genotypic characteristics and to provide a baseline for future medical, functional, and comparative studies. These isolates were selected to present a high genetic diversity; therefore, they cannot be used to imply their overall incidence of notified cases of salmonellosis. Yet, many conclusions still can be made with this fact in mind. 

Consistent with a previous study that examined 27 *S. enterica* serovar Schwarzengrund isolates from clinical sources [19], genome features, including genomic sizes, GC content, number of contigs, and number of coding sequences, were comparable with the results observed here, suggesting a consistency of WGS performance across laboratories. After whole-genome annotation, RAST server showed that nine subsystems (“photosynthesis”, “miscellaneous”, “nucleosides and nucleotides”, “cell division and cell cycle”, “motility and chemotaxis”, “secondary metabolism”, “fatty acids, lipids, and isoprenoids”, “nitrogen metabolism”, “sulfur metabolism”) were conserved and shared among all genomes, pinpointing that these may be core genome genes dedicated to metabolic functions and are needed to sustain bacterial life [20]. 

On the other hand, genes that varied from strain to strain were also observed in this study, indicating that these genes are accessory genomes and are important drivers to persist in a particular environment [21]. Nevertheless, minimal variations were observed among the *S. enterica* serovar Schwarzengrund genomes (<5%) in other subsystems, except for “Virulence, Disease and Defense”, “Phages, Prophages, Transposable elements, Plasmids”, and “Membrane Transport” subsystems. These >5% variations in these subsystems were found among the strains, suggesting that each strain had acquired different mobile elements to increase their resistance and virulence, which can confer itself a selective advantage under a selection process [22]. As numerous studies have shown that *Salmonella* could transfer virulence determinants to the cytoplasm of the infected host cell via bacterial outer membrane vesicles [23], identification of these accessory genomic elements, such as resistance and virulence, can help prepare responses more quickly to outbreaks of multiple antibiotic-resistant strains in healthcare settings.

Pathogens resistant to one or more clinically relevant antibiotics would necessitate new treatment strategies. Similar to previous studies [24], traditional first-line drugs such as ampicillin, chloramphenicol, and trimethoprim-sulfamethoxazole are ineffective under this investigation, and ciprofloxacin remains the most effective treatment. Within these strains, two isolates (SS02 and SS06) showed no resistance to chloramphenicol but carried *cml*A1-resistant genes. Moreover, 2 (SS16 and SS17) and 5 (SS04, SS14, SS15, SS16, and SS17) of the 23 *S. enterica* isolates predicted to be ampicillin- and ciprofloxacin-susceptible, respectively, were resistant. Hence, it is possible that these isolates contain an unknown gene or mutation that confers resistance. However, this assumption warrants further investigation.

Considering that all selected isolates exhibited multidrug resistance (resistant to three or more classes of antimicrobial) [25], and six of those were resistant to ciprofloxacin, this antibiotic resistance profile can be due to the prone usage of fluoroquinolone over traditional drugs and increased usage of fluoroquinolone in livestock for therapeutic and growth promotion purposes [26]. Previous research reported that the underlying mechanism for ciprofloxacin resistances may be caused by specific mutations in genes encoding DNA gyrase and topoisomerase IV that decrease quinolone sensitivity by weakening the interactions between quinolones and bacterial enzymes [27]. With the availability of WGS data and ResFinder’ sister database, PointFinder, two mutations were detected in gyrA (S83F and D87G) in strain SS20, confirming their associations with ciprofloxacin resistance [28].

Other than antimicrobial resistance genes, mobile genetic elements such as plasmids and integrons are also pivotal in the dissemination and persistence of antimicrobial resistance [29]. Earlier investigation has shown that plasmids, especially those from incompatible groups IncHI, IncF, IncP, and IncB/O, are the most frequently observed in multidrug-resistant *Salmonella enterica* serovar Typhi [30]. In another study, 902 *Salmonella* isolates representing 59 different serovars showed that IncFIB plasmid (also commonly known as ColV plasmids) was found to occur predominantly in serovar Kentucky (72.9% of isolates tested), followed by Typhimurium (15%), and Heidelberg (1.7%) [31]. Moreover, the acquisition of the IncFIB plasmid by *S. enterica* serovar Kentucky was found to increase its ability to colonize chicken cecum and cause significant extra-intestinal disease [31]. Hence, for the first time in this study, results showed that IncFIB(K) plasmid was the most prevalent replicon type (69.5%), followed by IncQ, Col440I, and Col440II within *S. enterica* serovar Schwarzengrund strains. According to prior studies, IncF plasmids often carry a *bla*_CTX-M_ gene [32] and IncQ plasmids often carry *str*AB, *tet*AR, and *sul*2 genes [33]. Despite the fact that Col plasmids encoded no known antimicrobial resistance genes, they seemed to be mobilized by co-resident conjugal plasmids, such as IncI1 and IncX [34]. As plasmids can be transferred between bacterial cells via horizontal gene transfer, determination of genetic determinant localized on plasmids may be required for further studies. 

Integrons are also capable of mobilizing antimicrobial resistance genes among bacteria. The results of the present study demonstrated that 95.6% of the selected strains contained Class I integron, which was higher than the 11–66% class I integron found among human and animal sources from previous work [35]. Other than SS21, each strain harbors a complete integron, which includes a 5′ conserved segment, a 3′ conserved segment, and a gene cassette that encodes antimicrobial resistance determinants [36]. In this study, up to six different gene cassettes were found that were associated with resistance to trimethoprim, streptomycin, tetracycline, sulfonamide, chloramphenicol, and gentamicin. Only one gene cassette, *dfr*A12*-aad*A, was consistent with the previous observations of gene cassettes found in *S. enterica* serovar Schwarzengrund isolates [37]. Other gene cassettes, including *dfr*A12-*aad*A-*cml*A, *dfr*A12-*aad*A-*cml*A-*sul*3, *aad*A-*cml*A-*sul*3, *dfr*A12-*aad*A-*cml*A-*tet*R-*tet*(A), and *dfr*A12-*aad*A-*aac*(6’)-Ib-cr-*cml*A-*sul*3, to our knowledge, were identified for the first time in *S. enterica* serovar Schwarzengrund isolates.

Regarding virulence factors, 8 out of 23 known SPIs [38] were detected, including SPI-1, SPI-2, SPI-3, SPI-4, SPI-5, SPI-9, SPI-13, and SPI-14, as well as C63PI, and these were detected in all isolates. The *Salmonella* SPI-1 (located within C63PI) and the SPI-2 encode type III secretion systems (T3SS), which are required for intestinal invasion and the production of enteritis [39]. The SPI-5 genes co-regulated with either SPI-1 or SPI-2 genes and encoded the effector proteins for both the T3SS encoded by SPI-1 and SPI-2 [40]. Recently, SPI-14 was found to play a role in the activation of SPI-1 genes and mediate bacterial invasion [41]. In addition to bacterial invasion, genes encoded on SPI-3 are important for gut colonization and intracellular survival [42,43], genes encoded on SPI-4 and SPI-9 are necessary for epithelial cell adhesion [44,45], and genes encoded on SPI-13 are pivotal for intracellular viability [46]. Nevertheless, the vast majority of these findings has been obtained in a mouse model and not in poultry, the latter of which represents a major reservoir of *Salmonella* for the human population [47]. Hence, more infection models using pigs, cattle, or poultry should be conducted in future studies to broaden our understanding of how SPIs contribute to *Salmonella* infection biology.

## 4. Materials and Methods

### 4.1. Bacterial Isolates

**Figure 1:**
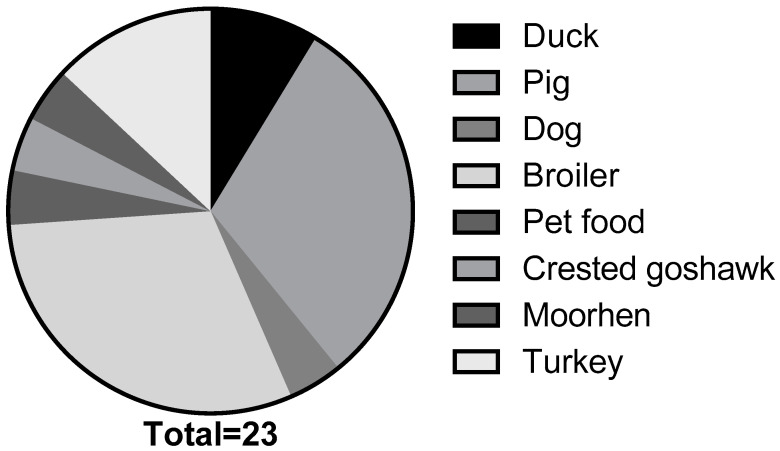
*Salmonella enterica* serovar Schwarzengrund isolated from animal and environmental sources collected between 2000 and 2018 (*n* = 23).

The *S. enterica* serovar Schwarzengrund strains analyzed in this study were collected between 2000 and 2018 (*n* = 23). These isolates were obtained from multiple sources, including duck (*n* = 2), pig (*n* = 7), dog (*n* = 1), broiler (*n* = 7), pet food (*n* = 1), crested goshawk (*n* = 1), moorhen (*n* = 1), and turkey (*n* = 3) (Figure 1). Recovered isolates were then subjected to serological tests, as previously described, for identification [48]. Prior to use, all strains were incubated overnight (14–16 h) on trypticase soy agar or in trypticase soy broth plated at 37 °C. 

### 4.2. Phenotypic Antimicrobial Susceptibility Testing

All antibiotics were acquired from Sigma-Aldrich Co. LLC (Saint Louis, MO, USA). For the 23 isolates collected, phenotypic susceptibility to 9 different antibiotics belonging to 6 antimicrobial classes (aminoglycoside: amikacin, tobramycin, gentamicin; phenicol: chloramphenicol; beta-lactam: ampicillin; fluoroquinolone: ciprofloxacin; folate pathway antagonist: sulfisoxazole, trimethoprim; tetracycline: tetracycline) was determined by broth microdilution. Classification as resistance was determined using breakpoints set by the Clinical and Laboratory Standards Institute [49].

### 4.3. Genome Library Preparation and Sequence Assembly

Genomic DNA was extracted using a DNeasy blood and tissue kit (Qiagen, CA, USA) according to the manufacturer’s instructions. DNA shearing was performed with a Misonix 3000 sonicator and checked by a DNA 1000 chip bioanalyzer (Agilent Technologies, Santa Clara, CA, USA). The DNA fragment length was between 180 and 200 base pairs (bp). Then, the sonicated DNA was end-repaired, A-tailed, and adaptor-ligated using the TruSeq DNA preparation kit (Illumina, San Diego, CA, USA) following the manufacturer’s guidelines. Libraries were sequenced using the NextSeq500 platform (Illumina, Inc., San Diego, CA, USA) with 150PE protocol. The average sequencing depth of the libraries was 944.4 MegaBase (~190X). Afterwards, the raw reads were trimmed and filtered using Trimmomatic software (version 0.36) developed by Bolger et al. [50]. Only reads with quality scores >18 and read sizes >10 were used for subsequent analysis. 

The trimmed reads of each sample were de novo assembled into contigs using SPAdes genome assembler (version 3.14.1) developed by Prjibelski et al. [51]. The assembled contigs of each sample were ordered, orientated, and joined into single scaffold using MeDuSa developed by Bosi et al. [52], based on the reference genome sequence (*S. enterica* subsp. enterica serovar Schwarzengrund strain CVM19633 of the EnsemblBacteria database (http://bacteria.ensembl.org/index.html), accessed on 12 August 2018). The WGS data used in this study were deposited to the NCBI database under BioProject accession number PRJNA635494.

### 4.4. Genome Annotation

Genomes of twenty-three *S. enterica* serovar Schwarzengrund strains were annotated using Rapid Annotation using a Subsystem Technology (RAST) server (https://rast.nmpdr.org/, accessed on 10 January 2021). Moreover, the identification of plasmid, antibiotics resistance gene, and *Salmonella* Pathogenicity Island (SPI) was performed by submitting the complete nucleotide sequence to PlasmidFinder, ResFinder, and SPIFinder, respectively, available at the Center for Genomic Epidemiology web server (https://cge.cbs.dtu.dk/services/, accessed on 10 January 2021). Annotation of integrons was conducted using the IntegronFinder [36], followed by protein Basic Local Alignment Search Tool (BLASTP) analysis. 

## 5. Conclusions

This study demonstrated that a considerable high antimicrobial resistance with a high virulence level of *Salmonella* was found from animal and environmental sources. For the first time, IncFIB plasmid was found to occur predominantly in *S. enterica* serovar Schwarzengrund isolates, which may increase its ability to colonize chicken cecum and cause extra-intestinal disease. Moreover, five different gene cassettes associated with resistance to trimethoprim, streptomycin, tetracycline, sulfonamide, chloramphenicol, and gentamicin were identified for the first time in *S. enterica* serovar Schwarzengrund isolates. As virulence and fitness can be encoded by mobile genetic elements, such as plasmids and integrons via horizontal gene transfer between *Salmonella*, these virulent species of bacteria can be acquired by humans via contaminated foods, thereby increasing the threat to public health. Hence, the availability of pathogen genome sequences, especially on *S. enterica* serovar Schwarzengrund, can not only help increase the reproducibility and accessibility of genomic analysis but can also support future surveillance of and epidemiological investigations into salmonellosis. With these baseline data, microbiologists and veterinarians can identify virulence traits of new emerging pathogens efficiently, and they can assist in the control of salmonellosis at the farm level. 

## Figures and Tables

**Table 1:** Genome annotation of twenty-three *Salmonella enterica* serovar Schwarzengrund strains.

Sample ID	Source	Size (Mb)	GC Content	Number of Contigs with Protein Encoding Genes	Number of Subsystems	Number of Coding Sequences	Number of RNA
SS01	Duck	4.81	52.1	163	367	4906	93
SS02	Pig	4.99	51.8	180	365	5176	104
SS03	Dog	4.81	52.2	12	366	4882	102
SS04	Pig	4.84	52.2	200	367	4979	96
SS05	Broiler	4.80	52.0	201	365	4955	104
SS06	Pig	4.79	52.1	129	367	4877	104
SS07	Broiler	4.64	52.2	130	363	4668	90
SS08	Broiler	4.73	52.2	178	367	4806	101
SS09	Pet food	4.64	52.2	138	363	4679	94
SS10	Broiler	4.72	52.2	148	367	4782	92
SS11	Broiler	4.83	52.1	28	366	4954	98
SS12	Crested Goshawk	4.95	52.0	26	371	5091	93
SS13	Moorhen	5.02	51.9	50	370	5208	91
SS14	Turkey	4.80	52.2	192	366	4907	89
SS15	Duck	4.79	52.2	173	366	4903	84
SS16	Pig	4.85	52.1	192	366	5030	87
SS17	Pig	4.85	52.1	186	366	5013	86
SS18	Turkey	4.76	52.2	148	367	4822	93
SS19	Turkey	4.72	52.2	143	367	4777	89
SS20	Pig	4.90	52.1	158	369	5028	102
SS21	Broiler	4.73	52.2	116	365	4793	102
SS22	Pig	4.78	52.0	127	367	4866	100
SS23	Broiler	4.74	52.2	21	366	4787	98

**Table 2:** RAST subsystem annotations of twenty-three *Salmonella enterica* serovar Schwarzengrund strains.

Sample ID	Cofactors, Vitamins, Prosthetic Groups, Pigments	Cell Wall and Capsule	Virulence, Disease and Defense	Potassium Metabolism	Phages, Prophages, Transposable Elements, Plasmids	Membrane Transport	Iron Acquisition and Metabolism	RNA Metabolism	Protein Metabolism	Regulation and Cell Signaling	DNA Metabolism	Dormancy and Sporulation	Respiration	Stress Response	Metabolism of Aromatic Compounds	Amino Acids and Derivatives	Phosphorus Metabolism	Carbohydrates
SS01	176	67	59	15	21	127	28	56	219	52	87	1	133	87	27	341	35	335
SS02	175	65	64	15	22	120	29	56	226	54	90	1	132	87	27	343	35	337
SS03	175	67	53	15	21	127	28	56	231	52	87	1	124	85	27	340	35	335
SS04	175	67	58	15	26	131	29	58	222	52	88	1	134	86	27	343	35	339
SS05	175	65	57	15	20	128	28	56	223	52	88	1	133	86	27	341	35	335
SS06	173	65	58	15	20	126	28	56	226	52	87	1	134	85	27	340	35	335
SS07	173	67	56	15	9	115	28	56	220	52	83	1	133	86	27	341	35	334
SS08	175	65	63	15	17	128	28	56	226	53	85	1	133	86	27	341	35	335
SS09	173	67	56	15	9	115	28	56	218	52	83	1	133	86	27	341	35	334
SS10	175	65	61	15	17	127	28	56	224	53	85	1	133	86	27	341	35	335
SS11	174	67	54	15	13	148	28	56	224	52	95	1	128	87	27	339	35	337
SS12	175	67	53	15	22	139	28	57	224	53	93	2	121	85	27	341	40	335
SS13	175	67	56	15	21	147	28	57	220	53	94	2	123	85	27	341	40	335
SS14	175	65	55	15	19	117	28	56	222	52	88	1	131	86	27	340	35	337
SS15	175	65	51	15	19	117	28	56	216	52	88	1	131	86	27	340	35	337
SS16	175	65	60	15	23	117	28	56	224	52	88	1	134	86	27	340	35	337
SS17	175	65	57	15	23	117	28	56	223	52	88	1	134	86	27	340	35	337
SS18	175	67	60	16	16	127	28	56	225	52	86	1	131	86	27	341	35	335
SS19	173	65	55	15	14	127	28	55	228	52	86	1	131	85	27	341	35	335
SS20	175	67	58	15	24	140	28	56	220	55	89	1	134	86	27	343	35	336
SS21	173	65	57	15	15	128	28	57	226	52	85	1	133	85	29	339	35	335
SS22	175	65	61	15	20	125	28	56	227	52	87	1	132	85	27	343	35	335
SS23	175	67	54	16	15	126	28	56	223	52	86	1	125	85	27	341	35	335

**Table 3:** Phenotypic and genotypic antibiotic resistance, including resistance genes harbored by 23 *Salmonella enterica* serovar Schwarzengrund.

	Phenotypic Resistance (ug/mL) ^a^										
Isolates	AMK	TB	GM	CL	AMP	CI	SXZ	TMP	TC	WGS-Predicted Resistance	Antimicrobial Resistance Genes
SS01	4	>128	64	>256	>256	<0.125	>1024	>256	128	AMK, TB, GM, CL, AMP, SXZ, TMP, TC	*aac(6’)-Iaa*, *aac(3)-IV*, *cmlA1*, *catA2*, *blaTEM-1B*, *sul1*, *sul2*, *sul3*, *dfrA12*, *tet(A)*
SS02	2	32	<0.5	8 ^b^	>256	<0.125	>1024	>256	128	AMK, TB, CL, AMP, SXZ, TMP, TC	*aac(6’)-Iaa*, *cmlA1*, *catA2*, *blaTEM-1B*, *sul3*, *dfrA12*, *tet(A)*, *tet(M)*
SS03	2	>128	64	>256	>256	<0.125	>1024	>256	128	AMK, TB, GM, CL, AMP, SXZ, TMP, TC	*aac(6’)-Iaa*, *aac(3)-IV*, *catA2*, *cmlA1*, *blaTEM-1B*, *sul2*, *sul3*, *sul1*, *dfrA12*, *tet(A)*
SS04	1	>128	32	>256	>256	32 ^b^	>1024	>256	256	AMK, TB, GM, CL, AMP, SXZ, TMP, TC	*aac(6’)-Iaa*, *aac(3)-IV*, *cmlA1*, *blaTEM-1B*, *sul3*, *sul1*, *dfrA12*, *tet(A)*
SS05	4	>128	32	64	>256	0.25	>1024	>256	128	AMK, TB, GM, CL, AMP, SXZ, TMP, TC	*aac(6’)-Iaa*, *aac(3)-IV*, *cmlA1*, *catA2*, *blaTEM-1B*, *sul2*, *sul1*, *sul3*, *dfrA12*, *tet(A)*
SS06	2	>128	32	4 ^b^	>256	<0.125	>1024	>256	128	AMK, TB, GM, CL, AMP, SXZ, TMP, TC	*aac(6’)-Iaa*, *aac(3)-IV*, *blaTEM-1B*, *sul1*, *dfrA12*, *tet(A)*
SS07	2	32	1	>256	1	<0.125	>1024	<0.5	1	AMK, TB, CL, SXZ	*aac(6’)-Iaa*, *catA2*, *cmlA1*, *sul3*, *sul2*
SS08	2	>128	32	64	>256	0.25	>1024	>256	128	AMK, TB, GM, CL, AMP, SXZ, TMP, TC	*aac(6’)-Iaa*, *aac(3)-IV*, *cmlA1*, *blaTEM-1B*, *sul3*, *sul1*, *dfrA12*, *tet(A)*
SS09	2	32	<0.5	>256	1	0.25	>1024	<0.5	1	AMK, TB, CL, SXZ	*aac(6’)-Iaa*, *catA2*, *cmlA1*, *sul3*, *sul2*
SS10	2	>128	64	64	>256	0.25	>1024	>256	128	AMK, TB, GM, CL, AMP, SXZ, TMP, TC	*aac(6’)-Iaa*, *aac(3)-IV*, *cmlA1*, *blaTEM-1B*, *sul1*, *sul3*, *dfrA12*, *tet(A)*
SS11	2	>128	16	256	>256	0.25	>1024	>256	128	AMK, TB, GM, CL, AMP, SXZ, TMP, TC	*aac(6’)-Iaa*, *aac(3)-IV*, *floR*, *cmlA1*, *blaTEM-1B*, *sul2*, *dfrA12*, *tet(A)*
SS12	2	>128	64	64	>256	0.25	>1024	>256	256	AMK, TB, GM, CL, AMP, SXZ, TMP, TC	*aac(6’)-Iaa*, *aac(3)-IV*, *cmlA1*, *blaTEM-1B*, *sul2*, *sul1*, *sul3*, *dfrA12*, *tet(A)*
SS13	2	>128	32	64	>256	0.25	>1024	>256	128	AMK, TB, GM, CL, AMP, SXZ, TMP, TC	*aac(6’)-Iaa*, *aac(3)-IV*, *cmlA1*, *blaTEM-1B*, *sul1*, *sul3*, *sul2*, *dfrA12*, *tet(A)*
SS14	1	>128	32	>256	2	32 ^b^	>1024	>256	256	AMK, TB, GM, CL, SXZ, TMP, TC	*aac(6’)-Iaa*, *aac(3)-IV*, *floR*, *cmlA1*, *sul3*, *sul1*, *dfrA12*, *tet(A)*
SS15	1	>128	32	>256	2	32 ^b^	>1024	>256	256	AMK, TB, GM, CL, SXZ, TMP, TC	*aac(6’)-Iaa*, *aac(3)-IV*, *cmlA1*, *floR*, *sul3*, *sul1*, *dfrA12*, *tet(A)*
SS16	1	>128	16	>256	>256 ^b^	32 ^b^	>1024	>256	2	AMK, TB, GM, CL, SXZ, TMP	*aac(6’)-Iaa*, *aac(3)-IV*, *floR*, *cmlA1*, *sul3*, *sul1*, *dfrA12*
SS17	1	>128	32	>256	256 ^b^	32 ^b^	>1024	>256	2	AMK, TB, GM, CL, SXZ, TMP	*aac(6’)-Iaa*, *aac(3)-IV*, *cmlA1*, *floR*, *sul3*, *sul1*, *dfrA12*
SS18	2	>128	32	64	>256	0.25	>1024	>256	128	AMK, TB, GM, CL, AMP, SXZ, TMP, TC	*aac(6’)-Iaa*, *aac(3)-IV*, *cmlA1*, *floR*, *blaTEM-1B*, *sul3*, *sul2*, *sul1*, *dfrA12*, *tet(A)*
SS19	2	32	<0.5	64	1	0.25	>1024	>256	128	AMK, TB, CL, SXZ, TMP, TC	*aac(6’)-Iaa*, *cmlA1*, *sul1*, *dfrA12*, *tet(A)*
SS20	8	>128	1	>256	>256	>64	>1024	>256	256	AMK, TB, GM, CL, AMP, CI, SXZ, TMP,	*aac(6’)-Ib-cr*, *aac(6’)-Iaa*, *aac(3)-IV*, *cmlA1*, *catA2*, *blaTEM-1B*, *sul1*, *sul3*, *sul2*, *dfrA12*, *tet(A)*
SS21	2	32	<0.5	4	>256	<0.125	>1024	>256	128	AMK, TB, AMP, SXZ, TMP, TC	*aac(6’)-Iaa*, *blaTEM-1B*, *sul1*, *dfrA12*, *tet(A)*
SS22	2	>128	<0.5	128	>256	<0.125	>1024	>256	128	AMK, TB, CL, AMP, SXZ, TMP, TC	*aac(6’)-Iaa*, *cmlA1*, *blaTEM-1B*, *sul1*, *sul3*, *dfrA12*, *tet(A)*
SS23	4	>128	32	256	>256	0.25	>1024	>256	128	AMK, TB, GM, CL, AMP, SXZ, TMP, TC	*aac(6’)-Iaa*, *aac(3)-IV*, *floR*, *cmlA1*, *blaTEM-1B*, *sul1*, *sul2*, *sul3*, *dfrA12*, *tet(A)*

^a^ AMK (amikacin); AMP (ampicillin); CL (chloramphenicol); CI (ciprofloxacin); GM (gentamicin); SXZ (sulfisoxazole); TC (tetracycline); TMP (trimethoprim); TB (tobramycin). ^b^ Phenotypic resistance was not correlated with the presence of antimicrobial resistance genes.

**Table 4:** Plasmids, integron, and *Salmonella* pathogenicity island of *Salmonella enterica* serovar Schwarzengrund from animal sources.

Isolates	Plasmids	Presence of Class I Integron	Gene Cassette Found on Integron	SPI
SS01	IncFIB(K)	1	*dfrA12 aadA cmlA sul3*	1, 2, 3, 4, 5, 9, 13, 14, C63PI
SS02	IncHI2, IncHI2A	1	*dfrA12 aadA cmlA*	1, 2, 3, 4, 5, 9, 13, 14, C63PI
SS03	IncFIB(K)	1	*dfrA12 aadA cmlA sul3*	1, 2, 3, 4, 5, 9, 13, 14, C63PI
SS04	IncFIB(K)	1	*dfrA12 aadA cmlA sul3*	1, 2, 3, 4, 5, 9, 13, 14, C63PI
SS05	IncFIB(K)	1	*dfrA12 aadA cmlA sul3*	1, 2, 3, 4, 5, 9, 13, 14, C63PI
SS06	Col(pHAD28), IncFIB(K)	1	*dfrA12 aadA*	1, 2, 3, 4, 5, 9, 13, 14, C63PI
SS07	-	1	*aadA cmlA sul3*	1, 2, 3, 4, 5, 9, 13, 14, C63PI
SS08	Col156, Col440II, IncFIB(K)	1	*dfrA12 aadA cmlA sul3*	1, 2, 3, 4, 5, 9, 13, 14, C63PI
SS09	-	1	*aadA cmlA sul3*	1, 2, 3, 4, 5, 9, 13, 14, C63PI
SS10	Col440II, IncFIB(K)	1	*dfrA12 aadA cmlA sul3*	1, 2, 3, 4, 5, 9, 13, 14, C63PI
SS11	Col(BS512), IncFIB(K), IncX1, IncL	1	*dfrA12 aadA cmlA sul2*	1, 2, 3, 4, 5, 9, 13, 14, C63PI
SS12	IncFIB(K)	1	*dfrA12 aadA cmlA sul3*	1, 2, 3, 4, 5, 9, 13, 14, C63PI
SS13	IncFIB(K), IncI2	1	*dfrA12 aadA cmlA sul3*	1, 2, 3, 4, 5, 9, 13, 14, C63PI
SS14	Col440I, IncFIB(K), IncQ1	1	*dfrA12 aadA cmlA tetR tet(A)*	1, 2, 3, 4, 5, 9, 13, 14, C63PI
SS15	Col440I, IncFIB(K), IncQ1	1	*dfrA12 aadA cmlA tetR tet(A)*	1, 2, 3, 4, 5, 9, 13, 14, C63PI
SS16	IncFIB(K), IncQ1	1	*dfrA12 aadA cmlA sul3*	1, 2, 3, 4, 5, 9, 13, 14, C63PI
SS17	IncFIB(K), IncQ1	1	*dfrA12 aadA cmlA sul3*	1, 2, 3, 4, 5, 9, 13, 14, C63PI
SS18	IncFIB(K)	1	*dfrA12 aadA cmlA sul3*	1, 2, 3, 4, 5, 9, 13, 14, C63PI
SS19	IncFIB(K)	1	*dfrA12 aadA cmlA*	1, 2, 3, 4, 5, 9, 13, 14, C63PI
SS20	IncFIB(K), IncFII(pCTU2), IncI1-I(Alpha)	1	*dfrA12 aadA aac(6’)-Ib-cr cmlA sul3*	1, 2, 3, 4, 5, 9, 13, 14, C63PI
SS21	IncFIB(K)	0	*-*	1, 2, 3, 4, 5, 9, 13, 14, C63PI
SS22	IncFIB(K)	1	*dfrA12 aadA cmlA tetR tet(A)*	1, 2, 3, 4, 5, 9, 13, 14, C63PI
SS23	IncFIB(K)	1	*dfrA12 aadA cmlA sul3*	1, 2, 3, 4, 5, 9, 13, 14, C63PI

## Data Availability

The WGS data used in this study were deposited to the NCBI database under BioProject accession number PRJNA635494.
